# Diabetes downregulates renal adenosine A_2A_ receptors in an experimental model of hypertension

**DOI:** 10.1371/journal.pone.0217552

**Published:** 2019-05-31

**Authors:** Daniela Patinha, Carla Carvalho, Carla Abreu, Olga M. Cunha, Mariana C. Mota, Joana Afonso, António Albino-Teixeira, Carmen Diniz, Manuela Morato

**Affiliations:** 1 Pharmacology and Therapeutics Unit, Department of Biomedicine, Faculty of Medicine, University of Porto, Porto, Portugal; 2 LAQV@REQUIMTE, Laboratory of Pharmacology, Department of Drug Sciences, Faculty of Pharmacy, University of Porto, Porto, Portugal; 3 MedInUP–Center for Drug Discovery and Innovative Medicines, University of Porto, Porto, Portugal; Escola Paulista de Medicina, BRAZIL

## Abstract

Studies on diabetic nephropathy rarely take into account that the co-existence of diabetes and hypertension is frequent and further aggravates the prognosis of renal dysfunction. Adenosine can activate four subtypes of adenosine receptors (A_1_, A_2A_, A_2B_ and A_3_) and has been implicated in diabetic nephropathy. However, it is not known if, in hypertensive conditions, diabetes alters the presence/distribution profile of renal adenosine receptors. The aim of this work was to describe the presence/distribution profile of the four adenosine receptors in six renal structures (superficial/deep glomeruli, proximal/distal tubules, loop of Henle, collecting tubule) of the hypertensive kidney and to evaluate whether it is altered by diabetes. Immunoreactivities against the adenosine receptors were analyzed in six renal structures from spontaneously hypertensive rats (SHR, the control group) and from SHR rats with diabetes induced by streptozotocyin (SHR-STZ group). Data showed, for the first time, that all adenosine receptors were present in the kidney of SHR rats, although the distribution pattern was specific for each adenosine receptor subtype. Also, induction of diabetes in the SHR was associated with downregulation of adenosine A_2A_ receptors, which might be relevant for the development of hypertensive diabetic nephropathy. This study highlights the adenosine A_2A_ receptors as a potential target to explore to prevent and/or treat early diabetes-induced hyperfiltration, at least in hypertensive conditions.

## Introduction

Diabetes Mellitus joins a group of metabolic diseases characterized by hyperglycemia and associated with high morbidity and mortality rates. It has reached epidemic proportions; approximately 422 million people worldwide have diabetes[1] and this number will continue to escalate, with predictions to rise up to 592 million by the year of 2035[2]. It is estimated that almost 1/3 of all diabetic patients will develop diabetic nephropathy[3], the prime cause of end-stage renal disease which, therefore, has a great impact on the use of health resources and associated costs[4]. In its initial stage, diabetic nephropathy is mainly characterized by glomerular hyperfiltration and hypertrophy, basal membrane thickening and mesangial matrix expansion that then progress to glomerulosclerosis, persistent proteinuria and decreased glomerular filtration rate (GFR)[3]. Early hyperfiltration is a good predictor for the development of end-stage renal disease[5].

Diabetes and hypertension independently contribute to the development of diabetic nephropathy and represent major causes of end-stage renal disease[4]. Also, the co-existence of these two chronic diseases is extremely frequent[6]. However, studies on diabetic nephropathy rarely take into account the co-existence of diabetes and hypertension, which further aggravates the prognosis of renal dysfunction[7, 8].

The mechanisms underlying diabetic nephropathy are multifactorial[3, 9] although still not fully characterized.

Adenosine regulates a wide range of physiological functions by activating four specific membrane receptor subtypes: A_1_, A_2A_, A_2B_ and A_3_, and has been implicated in diabetic nephropathy[10, 11]. In the kidney, adenosine is crucial for the maintenance of proper hemodynamics mainly through adenosine A_1_ receptor-mediated constriction of afferent arterioles and glomerular mesangial cells, and adenosine A_2A_ receptor-mediated vasodilation[12–15]. Moreover, adenosine A_1_ receptors also regulate tubular electrolyte transport and inhibit renin secretion[16, 17] while adenosine A_2A_ receptors contribute to maintain glomerular filtration[18] and mediate anti-inflammatory and immunosuppressive effects[19], although they stimulate renin release[20]. Adenosine A_2B_ receptors have been mainly associated with the production of vascular endothelial growth factor (VEGF)[21, 22] and stimulation of profibrotic and proinflammatory mediators[23]. The adenosine A_3_ receptors are known to induce mesangial cells apoptosis, which may represent a potential mechanism to limit glomerular mesangial expansion, an important histological feature of diabetic nephropathy[24].

In streptozotocin (STZ)-induced diabetes, it has been described an increased expression of adenosine A_1_ receptor mRNA but conflicting data has been reported concerning protein expression[21, 25, 26]. The adenosine A_2A_ receptor mRNA and protein levels are increased in the renal cortex of diabetic rats, and diabetes has also been associated with increased glomerular expression of adenosine A_2B_ receptors[21, 25, 26] and increased cortical levels of the adenosine A_3_ receptor protein[25]. The expression[25, 27–29] and function[11] of renal adenosine receptors is altered in normotensive animals with experimental diabetes when compared with normotensive controls. However, the coexistence of hypertension has never been addressed in this context. As so, the distribution profile of adenosine receptors is not fully described in hypertensive conditions neither is the impact of diabetes on a hypertensive background.

The aim of this work was to characterize the distribution profile of the four subtypes of adenosine receptors in renal structures of spontaneously hypertensive rats (SHR, our control group) and to evaluate whether it is altered by STZ-induced diabetes. We wanted to focus on early diabetic nephropathy, which is associated with hyperfiltration[3], a good predictor of end-stage renal disease[5]. As so, we decided to perform the experiments just 21 days after the induction of diabetes with STZ, since by this time the animals show hyperfiltration[11] and the associated renal disease mainly results from hyperglycemia and not of other putative confounding factors[30–32]. This study allowed us to describe, for the first time, the presence of all adenosine receptors in the kidney of SHR, and a downregulation of the adenosine A_2A_ receptors in SHR with STZ-induced diabetes.

## Material and methods

### Drugs

The following chemicals were used: STZ, Triton X-100 and DAB were obtained from Sigma Aldrich (Sintra, Portugal). The following primary antibodies were bought from Santa Cruz (Santa Cruz Biotechnology, CA, USA): rabbit polyclonal anti-A_1_, anti-A_2A_, anti-A_2B_, and anti-A_3_. The rabbit biotinylated secondary antibody and the avidin-biotin complex reagents (ABC) were obtained from Vectastain Elite ABC kit universal (Vector Laboratories, Burlingame, CA, USA). All reagents were of analytical grade.

### Animals and treatments

Male SHR animals (12 weeks; Charles River, Barcelona, Spain) were used. Animals had free access to water and food and were housed under controlled conditions of temperature (22°C), humidity (60%) and light-dark cycle (12 h/12 h). All animal procedures were performed according to the Portuguese DL n° 113/2013 and European Guidelines for humane and responsible animal care (European Directive 2010/63). All experiments were performed in accordance with the European Union guidelines for the protection of animals used for scientific purposes (Directive 2010/63/EU). Protocols are in accordance with the ARRIVE guidelines for reporting experiments[33] and were approved by the Committee on the Ethics of Animal Experiments of the Faculty of Pharmacy of the University of Porto (Permit Number 13/11/2013).

On day 0, SHR animals were randomly assigned to receive an intraperitoneal injection of STZ (65 mg/kg; SHR-STZ group, n = 4) or vehicle (sodium citrate buffer pH 4.5; SHR control group, n = 4). After 48 h, blood glucose concentration was determined using an autoanalyzer (Abbott Diabetes Care Ltd, Santa Clara, CA, USA) and animals with blood glucose concentration above 300 mg/dL were considered diabetic. On day 21, animals were anesthetized with pentobarbital sodium (50 mg/kg; i.p.) to minimize suffering, and the left kidney was excised and processed for immunohistochemistry. With this, the death of the anesthetized animals was ensured by exsanguination.

### Immunohistochemistry

The kidneys were fixed in 4% formaldehyde overnight, dehydrated in a graded series of ethanol followed by benzoyl, and embedded in paraffin. Sequential 4-μm-thick renal sections were obtained from each animal and mounted onto poly-L-lysine-coated slides.

Experiments were carried out in five batches using five levels, corresponding to 200 kidney sections for both SHR control and SHR-STZ groups.

Immunohistochemistry was performed as previously described[34] with some modifications. Briefly, sections were dewaxed with toluene and rehydrated in a graded series of ethanol at room temperature (RT). Endogenous peroxidase was blocked using 3% hydrogen peroxide (H_2_O_2_) and non-specific protein binding was blocked with 2% serum in phosphate-buffered saline [PBS (g/L): 8g NaCl; 0,201g KCl; 0,191g KH_2_PO_4_; 0,765g Na_2_HPO_4_.2H_2_O]. Sections were then incubated with rabbit primary polyclonal antibodies, anti-A_1_ (1:50 dilution), anti-A_2A_ (1:250 dilution), anti-A_2B_ (1:50 dilution), and anti-A_3_ (1:250 dilution). The specificity of these primary antibodies has been previously tested by other authors by immunoprecipitation of the protein or knockdown using siRNA[35–38] and by our group in SHR animals[39], by pre-adsorbing individual primary antibody with a tenfold excess of its respective blocking peptides, overnight, at 4°C.

**Fig 1:**
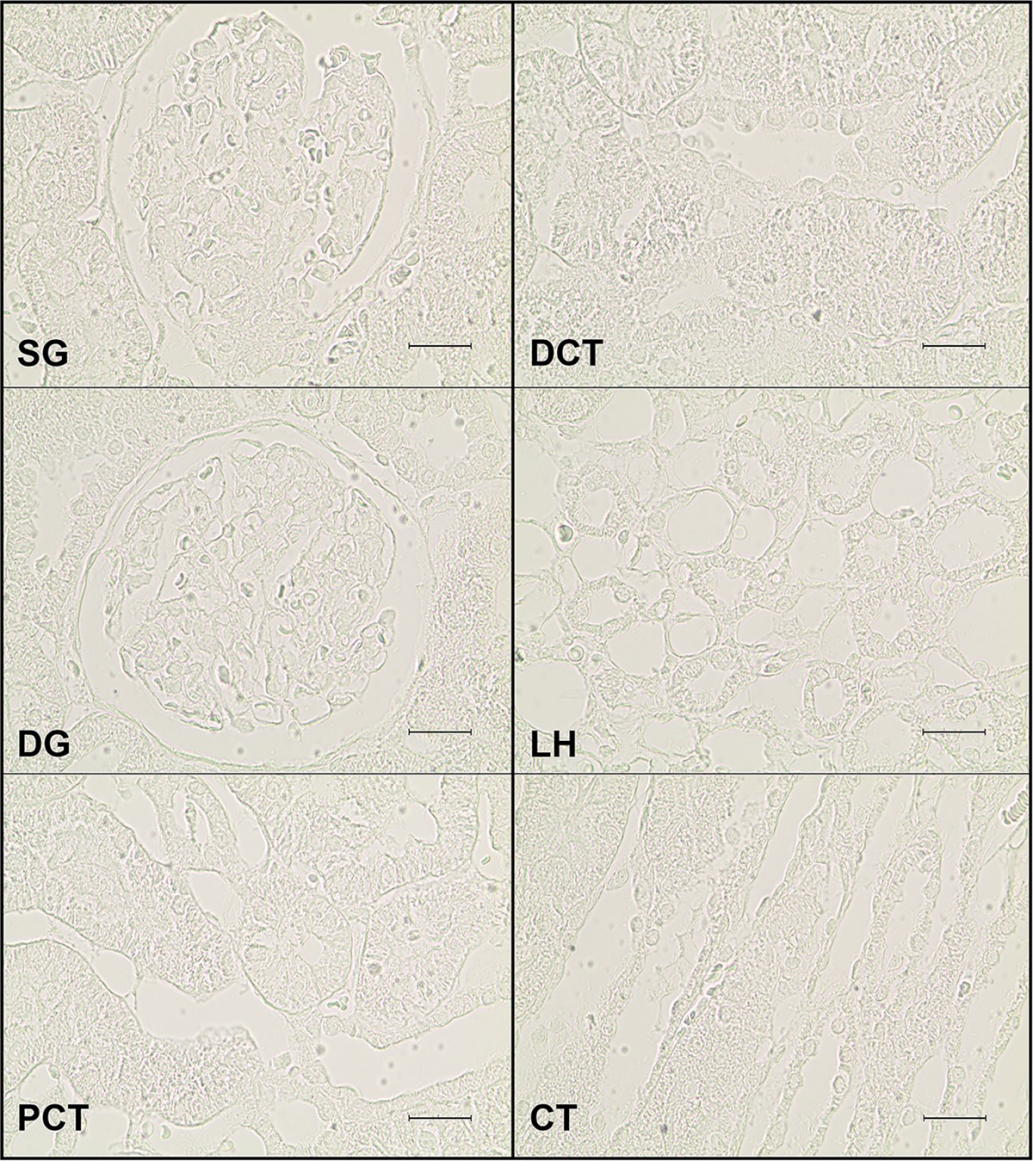
Negative controls of kidney transversal sections from SHR animals. Representative photomicrographs of kidney sections from 4 SHR rats incubated in parallel with 10% normal horse serum instead of the primary antibody to assess the level of background ascribed to nonspecific binding of the secondary antibody. A clean background was obtained in superficial (SG) and deep (DG) glomeruli, proximal (PCT) and distal (DCT) convoluted tubule, loop of Henle (LH) or collecting tubule (CT). Scale bar: 20 μm.

Incubation with individual adenosine receptor primary antibodies was done overnight, at 4°C, in a humidified chamber. Sections were subsequently rinsed in PBT and incubated with a biotinylated anti-rabbit secondary antibody (1:125 dilution in PBT) for 1h, at RT. Sections were then rinsed in PBT and incubated with avidin-biotin complex reagent (ABC) for 1h, at RT. After washing with PBS, sections were incubated with 3,3-diaminobenzidine tetrahydrochloride (DAB) activated with H_2_O_2_, used as a chromophore. The reaction was stopped by washing sections with distilled water. Finally, sections were dehydrated in a graded series of ethanol and xylene, and mounted with Eukitt mounting medium. For negative controls (controls for non-specific binding of secondary antibody) primary antibodies were omitted (Fig 1).

### Imaging

Micrographs of each immunostained section were acquired using a CDC camera (Leica DFC295, Leica Microsystems, Heerbrugg Switzerland) mounted on the microscope Nikon Eclipse E400 (objective 20x/0.5; ∞/0.17; WD 2.1; Nikon Corporation, Tokyo, Japan), using software Leica Microsystems software version 3.5.0 (Leica Microsystems, Heerbrugg, Switzerland). Illumination conditions of the bright field optics and camera exposure were maintained constant throughout the acquisition of all tissue sections, including control negative sections. Acquired images (24 bit, 8 bits/color) with resolution of 3072x2304 pixels corresponded to 655.36x491.52 μm area on the original histological section (1 pixel = 0.21 μm, a calibration micrometer slide was used to convert pixels into micrometers). These images were used both for qualitative analysis and histomorphometry.

### Histomorphometry

Histomorphometric analysis has been previously described as a valid methodology[40–42] and can be as effective as PCR or WB for quantitative measurements. Therefore, quantitative analysis and processing of digital images from DAB-immunostained sections were assessed using the SACAIA method and the PAQI software (CEMUP, Porto, Portugal), as previously described[43, 44]. Briefly, from RGB (red, green, blue) digital color images, only the blue component was selected for analysis, due to its higher contrast. RGB color images from DAB-immunostained sections (immunostained with anti-A_1_, anti-A_2A_, anti-A_2B_ or anti-A_3_ antibodies) were converted into their blue component and the renal structures were isolated. Boundaries were delineated to extract the object of interest and to set thresholds for automated DAB-staining segmentation using image analysis. As immunohistochemistry can provide detailed information concerning the location/presence/area of immunostaining, to make the analysis more comprehensive, we evaluated the expression on six different renal structures: superficial (SG) and deep (DG) glomeruli, proximal (PCT) and distal (DCT) collecting tubules, loop of Henle (LH) and collecting tubule (CT).

To determine differences between stained and non-stained tissue, negative control sections were imaged with the same microscope illumination and camera operating conditions, and the average of stained level was determined: a value of 171 for a maximum of 255. This average value was used for threshold segmentation of the stained areas of each kidney structure. The level of immunostaining was obtained by quantifying the fraction of the tissue that stained with DAB (stained fractional area) using digital images of DAB-labeled immunostains from kidney sections.

### Statistical analysis

Immunostaining was expressed as percentage of the tissue total area. Results were presented as median and 25^th^-75^th^ percentiles (P25-P75); n denotes the number of animals used in each group. In the SHR control group and for each adenosine receptor subtype, the differences in immunostaining observed between the different renal structures analyzed were compared with Kruskal-Wallis with Dunn's multiple comparisons test. Also, for each renal structure studied (SG, DG, PCT, DCT, LH and CD), the Mann-Whitney test was used to compare the immunostaining against each receptor between SHR control and SHR-STZ groups. In any case, GraphPad Prism 7 software was used for the statistical analysis and a p value <0.05 was considered significant.

## Results

The presence/distribution profile of the adenosine receptor subtypes A_1_, A_2A_, A_2B_ and A_3_ was characterized in kidney nephron of the SHR (our control group; a well-known hypertensive animal model[45, 46]) and compared to that observed in the SHR with STZ-induced diabetes.

### Distribution profile of adenosine receptors along the renal structures of the SHR group

**Fig 2:**
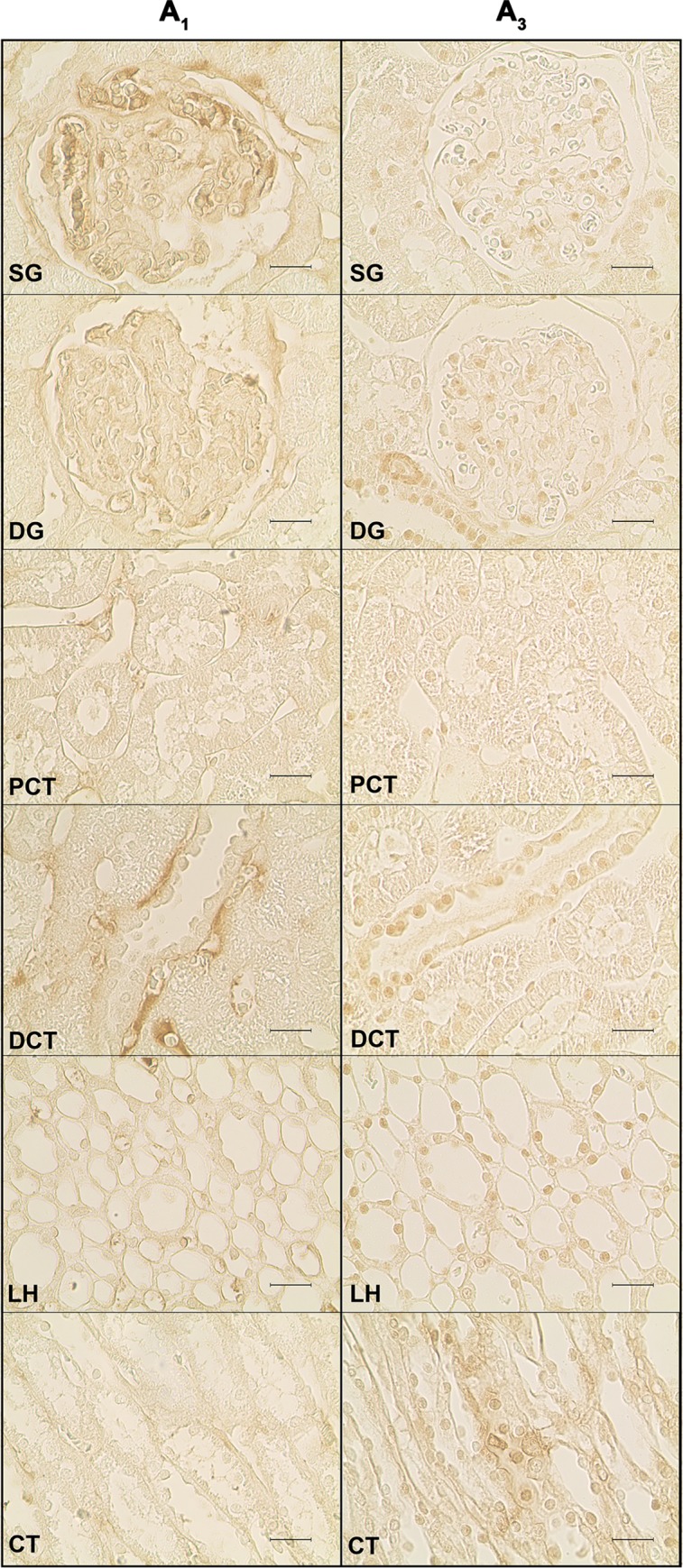
Immunoreactivity against the adenosine A_1_ and A_3_ receptors in the SHR control group. Representative photomicrographs of kidney sections from 4 SHR control rats incubated with a primary antibody against the adenosine A_1_ (left panels) and A_3_ (right panels) receptors. The six renal structures studied in separate are represented: superficial (SG) and deep glomeruli (DG), proximal (PCT) and distal (DCT) convoluted tubule, loop of Henle (LH) and collecting tubule (CT). Scale bars: 20 μm.

**Fig 3:**
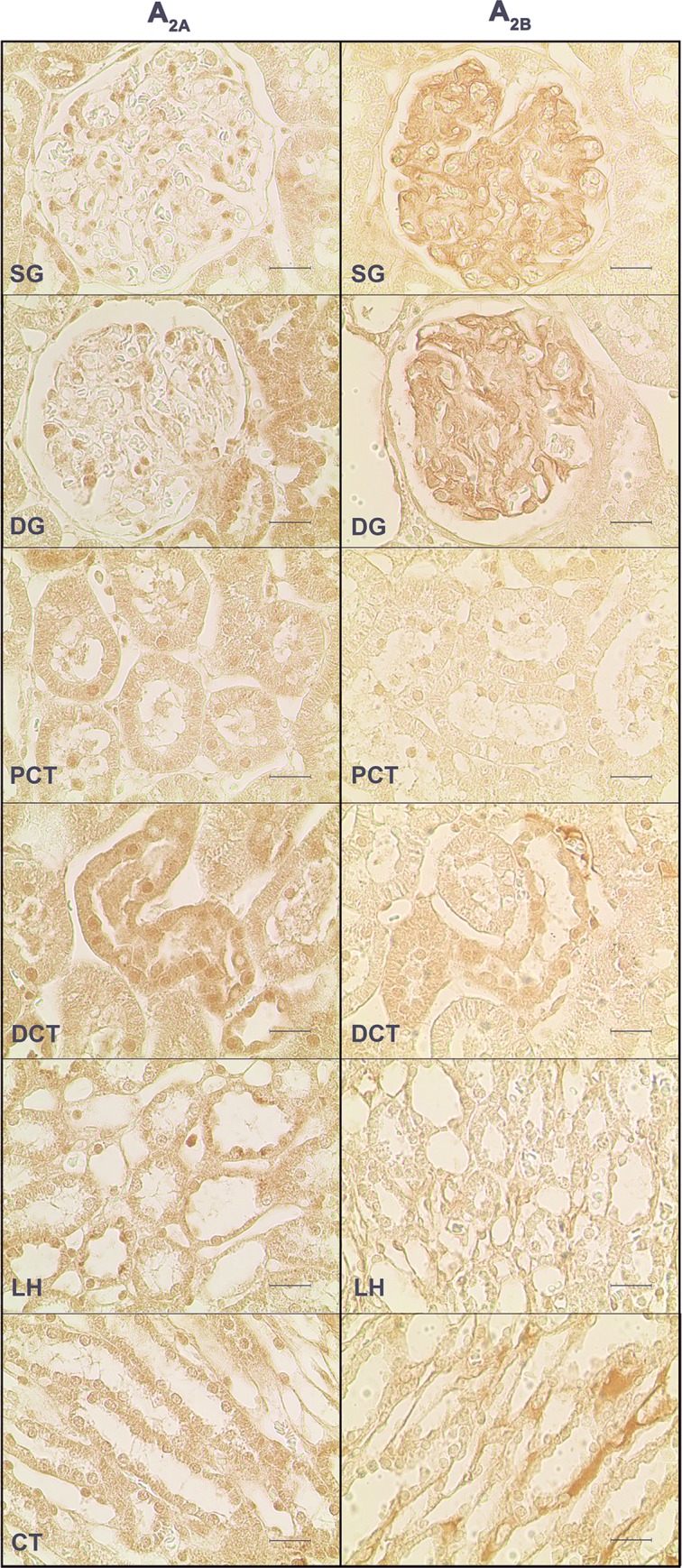
Immunoreactivity against the adenosine A_2A_ and A_2B_ receptors receptors in the SHR control group. Representative photomicrographs of kidney sections from 4 SHR control rats incubated with a primary antibody against the adenosine A_2A_ (left panels) and A_2B_ (right panels) receptors. The six renal structures studied in separate are represented: superficial (SG) and deep glomeruli (DG), proximal (PCT) and distal (DCT) convoluted tubule, loop of Henle (LH) and collecting tubule (CT). Scale bars: 20 μm.

Representative images of the immunoreactivity observed against each of the four adenosine receptor subtypes studied (A_1_, A_2A_, A_2B_ and A_3_) in the renal structures of SHR control rats are depicted in Fig 2 and Fig 3. Immunoreactivity against the four adenosine receptor subtypes was observed in all the kidney structures studied: SG, DG, PCT, DCT, LH and CT.

**Fig 4:**
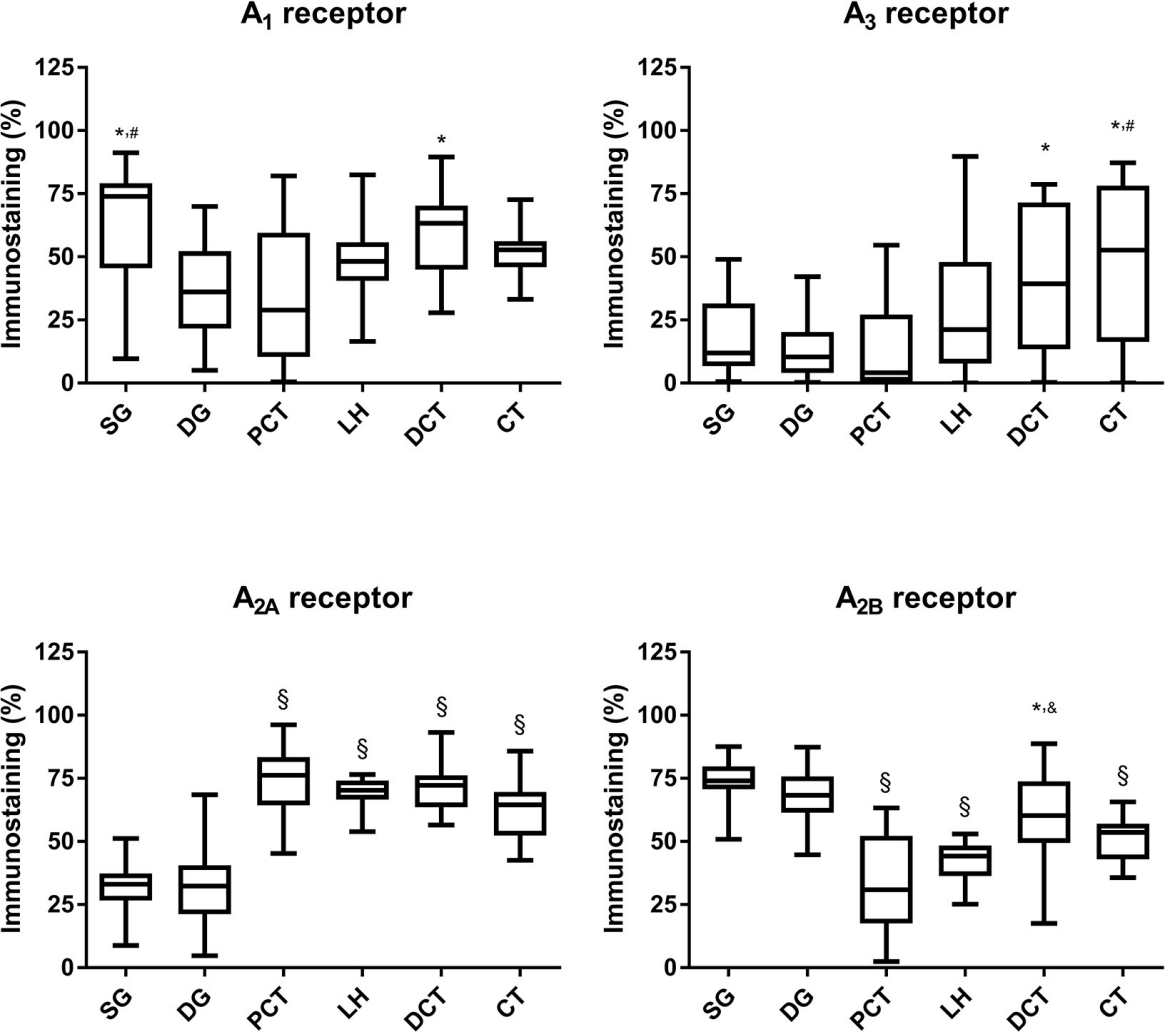
Quantitative immunostaining for A_1_, A_2A_, A_2B_, and A_3_ adenosine receptors in the renal structures from SHR control rats. Quantitative analysis of the immunostaining (staining fractional area in percentage of the tissue total area; using the SACAIA method) for the adenosine A_1_, A_2A_, A_2B_, and A_3_ receptors in the six renal structures from SHR control rats. Superficial (SG) and deep (DG) glomeruli, proximal (PCT) and distal (DCT) convoluted tubule, loop of Henle (LH) and collecting tubule (CT). Values are median and 25th-75th percentiles (P25-P75) from 4 rats. * p<0.05 *vs* corresponding PCT; ^#^ p<0.05 *vs* corresponding DG; ^§^ p<0.05 *vs* corresponding SG and DG; ^&^ p<0.05 *vs* corresponding LH.

In SHR control animals, the SG was the structure that showed the highest immunoreactivity against the adenosine A_1_ receptor (Fig 2, left panels and Fig 4). The distribution of adenosine A_1_-receptor immunoreactivity was variable along glomerular cells, with higher immunoreactivity in mesangial cells and lower in podocytes. The parietal layer of the Bowman's capsule also showed some adenosine A_1_ receptor immunoreactivity. Adenosine A_1_ receptor immunoreactivity was also present in the other kidney structures (Fig 2, left panels and Fig 4). In the DG, it was located mainly in mesangial cells; the PCT was the renal structure presenting less adenosine A_1_ receptor immunoreactivity (Fig 2, left panels and Fig 4); in DCT, LH and CT, adenosine A_1_ receptor immunoreactivity was distributed along the basal border of the tubular cells, mostly located in the vasa recta (Fig 2, left panels and Fig 4).

Immunoreactivity against the adenosine A_2A_ receptor was lower in the glomeruli than in the renal tubular structures (PCT, DCT, LH and CT) (Fig 3, left panels and Fig 4). SG and DG presented similar levels of adenosine A_2A_ receptor immunoreactivity, which was found to be distributed mainly in mesangial cells although it was also observed in both the parietal and visceral (podocytes) layers of the Bowman's capsule (Fig 3, left panels). In tubular structures, adenosine A_2A_ receptor immunostaining was observed in the nuclei and membrane of tubular cells.

Conversely, the kidney structures with higher adenosine A_2B_ receptor immunoreactivity were the glomeruli (SG and DG) followed by DCT and, much less, the other tubular structures (PCT, LH and CT) (Fig 3, right panels and Fig 4). Among glomeruli, the immunoreactivity against the adenosine A_2B_ receptor was distributed between mesangial and podocytes, but was almost absent in the parietal layer of the Bowman's capsule (Fig 3, right panels). In the LH, immunoreactivity against the adenosine A_2B_ receptor was present in the vicinity of the basal border of cells, in the vasa recta (Fig 3, right panels).

Adenosine A_3_ receptor immunoreactivity was weaker than that observed for the other adenosine receptors in every kidney structure studied (Fig 2, right panels). Immunoreactivity against the adenosine A_3_ receptor was located both in the nuclei and membrane of the cells. In the glomeruli (both SG and DG) it was mainly found within the nuclei of mesangial cells and in the Bowman's capsule, where although sparse, it was primarily present in the parietal layer (Fig 2, right panels). As for the other kidney structures, the PCT showed the lowest adenosine A_3_ receptor immunoreactivity while DCT and CT were the structures presenting the highest adenosine A_3_ receptor immunoreactivity (Fig 2, right panels and Fig 4).

### Distribution profile of adenosine receptors along the renal structures of the SHR-STZ group

**Fig 5:**
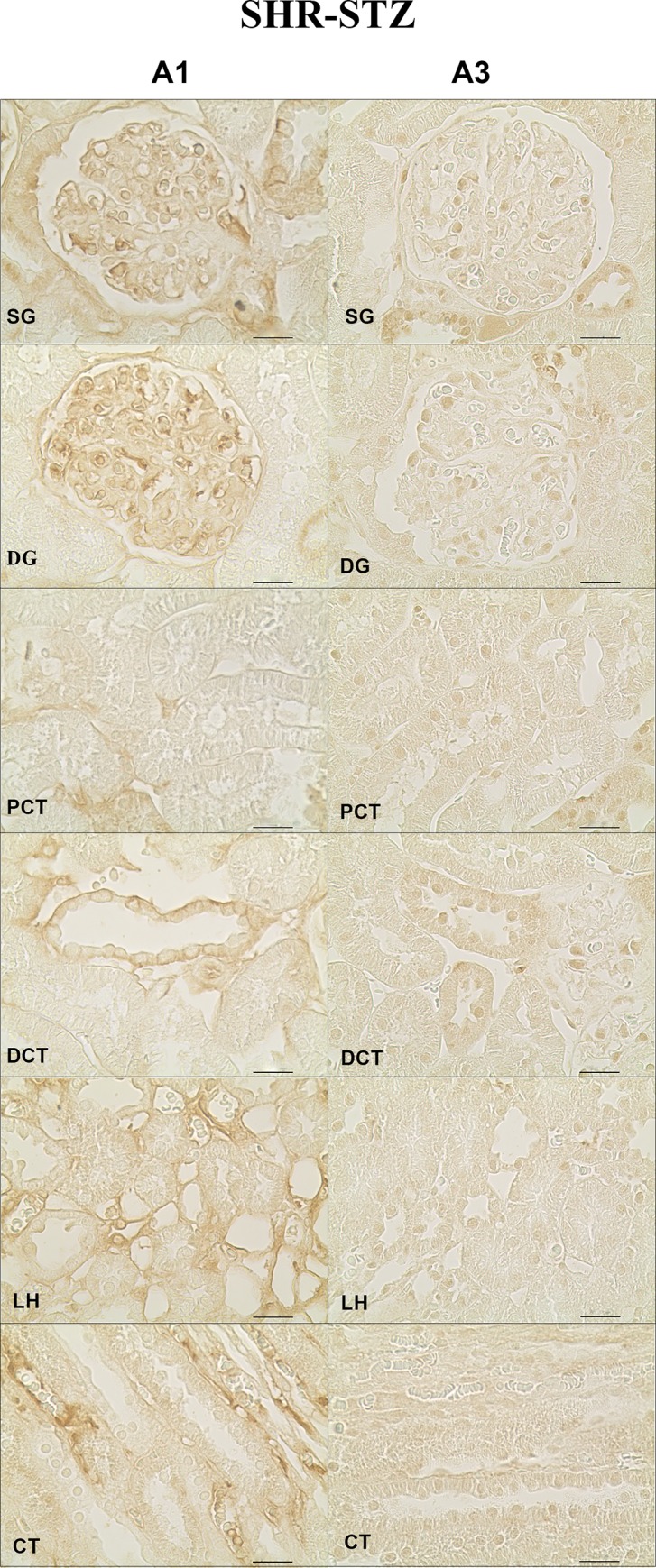
Immunoreactivity against the adenosine A_1_ and A_3_ receptors in rats simultaneously having hypertension and diabetes. Representative photomicrographs of kidney sections from 4 SHR-STZ rats incubated with a primary antibody against the adenosine A_1_ (left panels) and A_3_ (right panels) receptors. The six renal structures studied in separate are represented: superficial (SG) and deep glomeruli (DG), proximal (PCT) and distal (DCT) convoluted tubule, loop of Henle (LH) and collecting tubule (CT). Scale bars: 20 μm.

**Fig 6:**
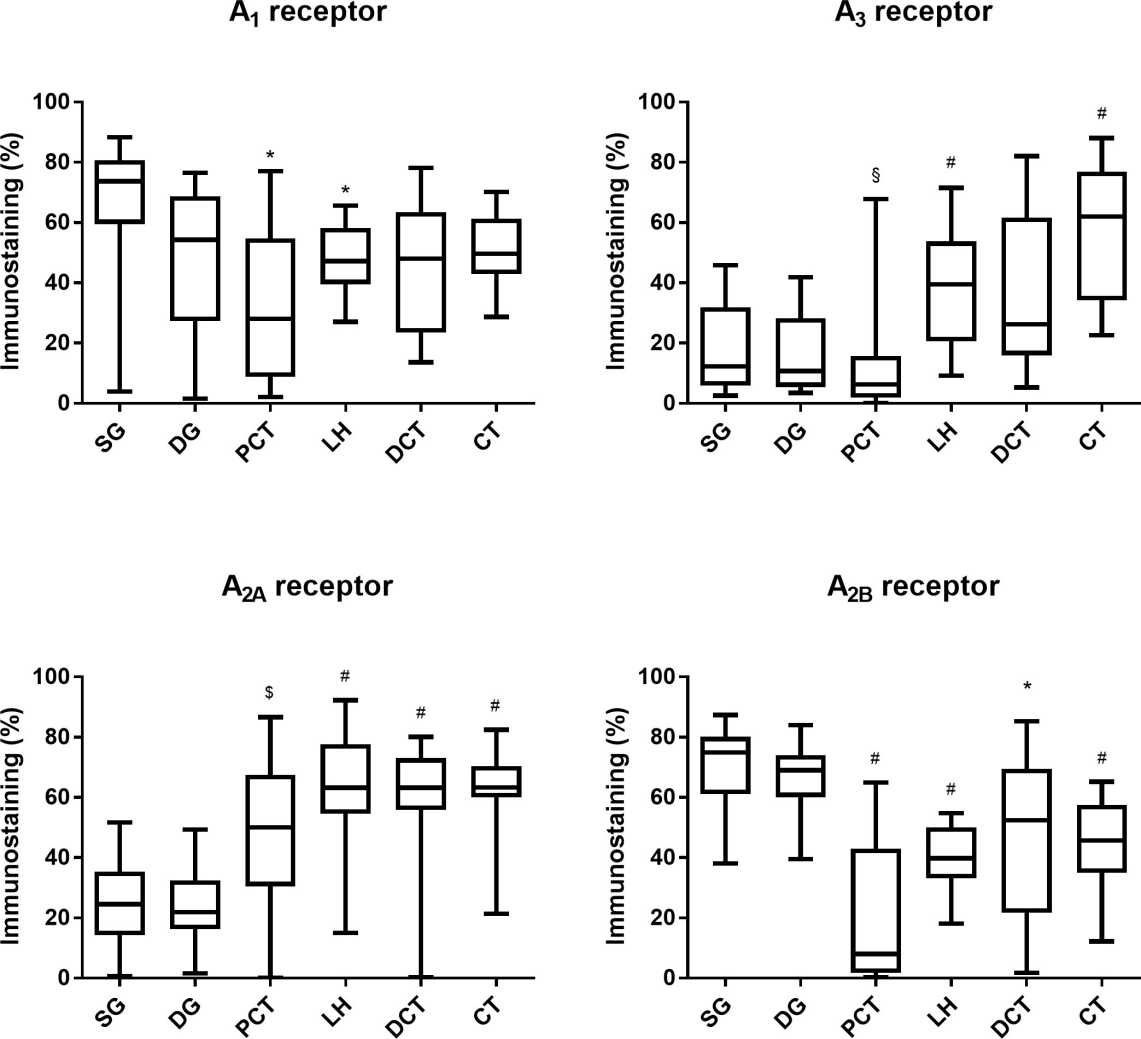
Quantitative immunostaining for A_1_, A_2A_, A_2B_, and A_3_ adenosine receptors in the renal structures from SHR-STZ rats. Quantitative analysis of the immunostaining (staining fractional area in percentage of the tissue total area; using the SACAIA method) for the adenosine A_1_, A_2A_, A_2B_, and A_3_ receptors in the six renal structures from SHR-STZ rats. Superficial (SG) and deep (DG) glomeruli, proximal (PCT) and distal (DCT) convoluted tubule, loop of Henle (LH) and collecting tubule (CT). Values are median and 25th-75th percentiles (P25-P75) from 4 rats. * p<0.05 *vs* corresponding PCT; ^#^ p<0.05 *vs* corresponding DG; ^§^ p<0.05 *vs* corresponding SG and DG; ^&^ p<0.05 *vs* corresponding LH.

**Fig 7:**
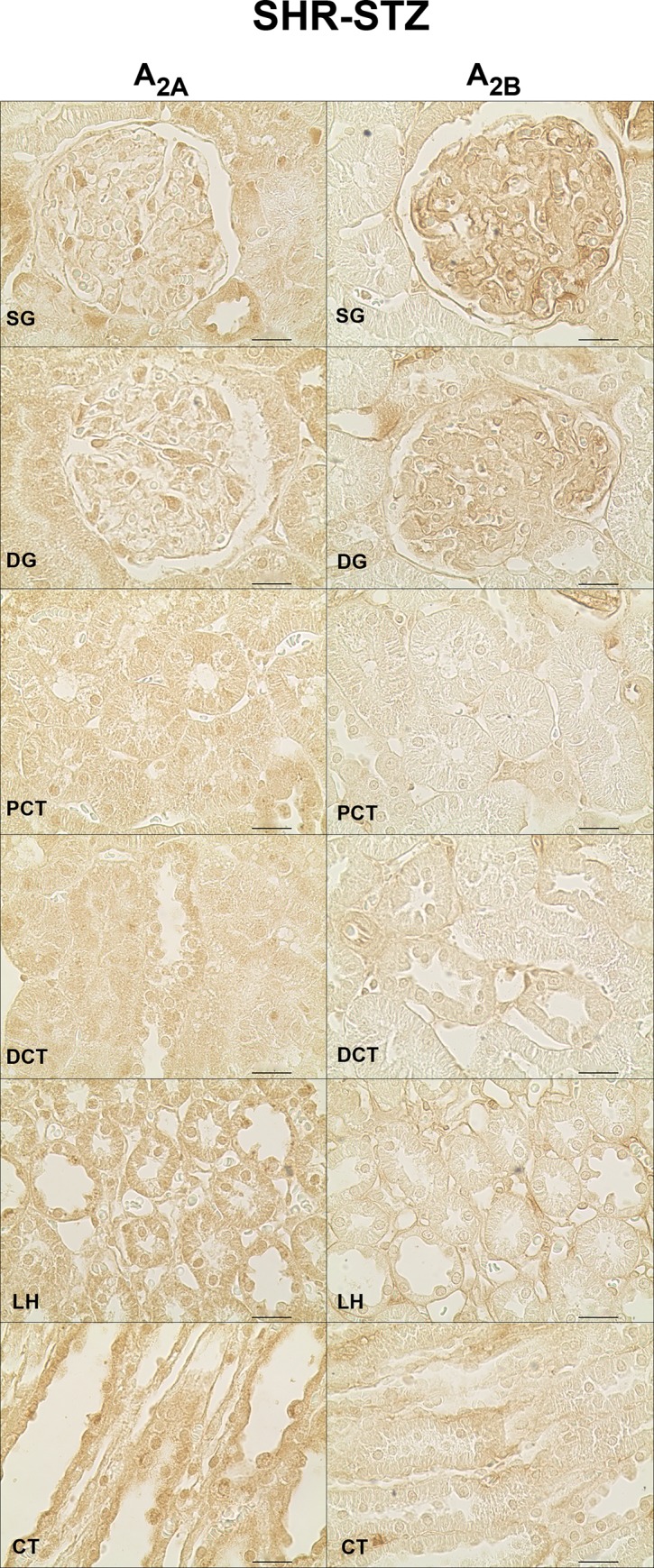
Immunoreactivity against the adenosine A_2A_ and A_2B_ receptors in rats simultaneously having hypertension and diabetes. Representative photomicrographs of kidney sections from 4 SHR-STZ rats incubated with a primary antibody against the adenosine A_2A_ (left panels) and A_2B_ (right panels) receptors. The six renal structures studied in separate are represented: superficial (SG) and deep glomeruli (DG), proximal (PCT) and distal (DCT) convoluted tubule, loop of Henle (LH) and collecting tubule (CT). Scale bars: 20 μm.

In SHR-STZ animals, immunoreactivity against the four adenosine receptor subtypes was also observed in all the kidney structures studied: SG, DG, PCT, LH, DCT and CT. The SG was the structure that showed the highest immunoreactivity against the A_1_ receptor, the difference being statistically different for PCT and LH (Fig 5, left panels and Fig 6). Also, the glomeruli (both SG and DG) were the kidney structures with more marked A_2B_ immunoreactivity (Fig 7, right panels and Fig 6). Immunoreactivity against the adenosine A_2A_ receptor was similar between SG and DG but it was lower in the glomeruli than in the renal tubular structures (Fig 6 and Fig 7, left panels). Likewise, immunoreactivity against the adenosine A_3_ receptor was also similar between SG and DG and it was lower in the glomeruli than in the tubular structures, namely the LH and CT (Fig 5, right panels and Fig 6). The immunoreactivity against the adenosine A_3_ receptor was also very low in the PCT compared with the other renal tubular structures (Fig 5, right panels and Fig 6). Generally, A_3_ receptor immunoreactivity was weaker than that observed for the other adenosine receptors (Fig 5, right panels and Fig 6).

### STZ-induced diabetes altered the renal distribution profile of adenosine receptors of the SHR

We found that adenosine A_1_ receptor immunoreactivity was numerically higher in DG and lower in DCT from SHR-STZ (Table 1) comparatively to the correspondent structures of SHR control animals. The adenosine A_2A_ receptor immunoreactivity found in DG, PCT and DCT of SHR-STZ animals was lower than that found in the correspondent structures of SHR controls (Table 1). Concerning the immunoreactivities against adenosine A_2B_ (Table 1) and A_3_ (Table 1) receptors, there were no differences between renal structures of SHR-STZ and SHR control animals except for a tendency for a lower immunoreactivity against the adenosine A_2B_ receptor in the PCT of SHR-STZ.

**Table 1:** Immunostaining (% of the tissue total area) for A_1_, A_2A_, A_2B_, and A_3_ adenosine receptors in the renal structures from SHR control and SHR-STZ groups.

		SHR control	SHR-STZ	p
Adenosine A_1_ receptor				
	SG	73.92 (45.52–79.07)	73.73 (59.53–80.82)	0.772
	DG	36.05 (21.57–52.23)	54.31 (27.32–68.69)	0.081
	PCT	28.95 (10.39–59.51)	28.05 (8.82–54.82)	0.772
	DCT	63.23 (44.93–70.23)	48.04 (23.43–63.49)	0.079
	LH	48.23 (40.44–55.78)	47.24 (39.53–58.24)	0.982
	CT	52.81 (45.86–56.19)	49.71 (42.95–61.29)	0.959
Adenosine A_2A_ receptor				
	SG	32.15 (26.46–37.37)	24.03 (13.09–36.5)	0.115
	DG	33.32 (22.75–40.90)	21.26 (15.82–31.88)	0.032
	PCT	75.98 (63.23–82.24)	50.06 (31.65–68.38)	0.001
	DCT	72.26 (63.05–76.99)	63.22 (55.90–72.37)	0.049
	LH	70.21 (66.60–74.07)	62.64 (53.59–76.94)	0.200
	CT	64.73 (51.12–70.26)	63.39 (60.15–72.25)	0.392
Adenosine A_2B_ receptor				
	SG	74.06 (70.70–79.73)	74.94 (61.10–80.16)	0.984
	DG	68.32 (61.39–75.78)	69.02 (60.16–74.04)	0.855
	PCT	30.84 (17.53–52.23)	8.06 (1.82–42.92)	0.078
	DCT	60.22 (49.48–73.84)	52.38 (21.83–69.54)	0.102
	LH	44.27 (36.32–48.34)	39.81 (33.22–50.03)	0.525
	CT	53.60 (42.96–57.01)	45.74 (34.95–57.54)	0.262
Adenosine A_3_ receptor				
	SG	11.89 (6.75–31.51)	12.18 (5.88–31.87)	0.886
	DG	10.32 (4.02–20.26)	10.73 (5.48–28.27)	0.568
	PCT	4.14 (0.54–27.16)	6.32 (1.91–15.77)	0.539
	DCT	39.32 (13.38–71.46)	26.26 (15.99–61.62)	0.822
	LH	53.78 (26.42–82.10)	43.55 (33.41–63.19)	0.140
	CT	52.67 (16.27–78.15)	62.05 (34.18–76.89)	0.328

Values are median (P25-P75) from 4 rats. SG = superficial glomeruli; DG = deep glomeruli; PCT = proximal convoluted tubule; DCT = distal convoluted tubule; LH = loop of Henle; CT = collecting tubule.

## Discussion

The results of the present study reveal, for the first time, a differential expression and distribution pattern of the four adenosine receptor subtypes along the nephron of the SHR. Additionally and also innovative, this study uncovers a downregulation of renal adenosine A_2A_ receptors caused by STZ-induced diabetes in hypertensive conditions.

### Expression of adenosine receptors along the nephron in the SHR control group

Our results indicate that adenosine A_1_ receptors are mostly present in SG and DCT, while its presence in DG and in the other tubular structures studied is less marked and similar between them. This differential staining between SG and DG was only found for this adenosine receptor subtype and probably reflects the adenosine A_1_ receptor-mediated afferent vasoconstriction that is crucial for renal autoregulation of blood flow that predominates in the renal superficial cortex[47]. In our study, with hypertensive rats, adenosine A_1_ receptor was more markedly present in mesangial cells, which contract[12] and contribute to renal autoregulation of blood flow[48]. Differently, in a study with normotensive rats, the adenosine A_1_ receptor was described in mesangial cells[27] but mostly in the epithelial cells of the glomeruli[25]. The immunolocalization and/or mRNA expression of adenosine A_1_ receptors in the normal kidney has already been reported in all renal structures[22, 25, 27–29, 49]. Its presence in the PCT is not so consensual[28, 29, 49–52]. We confirm the presence of the adenosine A_1_ receptor in PCT although this was the less marked renal structure.

In our study with SHR animals, the presence of adenosine A_2A_ receptor was more marked in the renal tubular structures than the glomeruli. In the renal tubules, adenosine A_2A_ receptors inhibit tubular sodium reabsorption in the distal nephron[53], thus causing diuretic and natriuretic effects, and increase distal Mg^2+^ uptake[54]. Again differently, in the normotensive kidney, adenosine A_2A_ receptors have been described to be mainly located in the vasculature and the glomeruli, with lower expression reported within the tubular structures of cortex and medulla[29], which is consistent with the vasodilatory role of the adenosine A_2A_ receptor, especially in the deep renal cortex and medulla[13].

Similarly to what was observed for the adenosine A_1_ receptor, the adenosine A_2B_ receptor was markedly present in glomeruli and DCT when compared with the other renal tubular structures. Adenosine A_2B_ receptors increase VEGF production[21, 22] and release[22] in glomerular mesangial cells and podocytes. Although the mRNA for the adenosine A_2B_ receptor is mostly found in the DCT[29], we also found adenosine A_2B_ receptors in CT, where they stimulate chloride secretion[55]. PCT and LH were the structures with the lowest immunoreactivity against the adenosine A_2B_ receptor and their putative function has not been studied yet.

Immunoreactivity against the adenosine A_3_ receptor was the weakest of all adenosine receptors studied. Still, structures of the distal nephron were more markedly stained. Accordingly, mRNA for the adenosine A_3_ receptor has already been found in the whole kidney of normotensive Wistar rats although with a low expression[29], but so far, no distinct intrarenal localization has been reported. Also, very little is known about this receptor and its functions along the nephron. Even though it has been suggested that, in basal physiological conditions, adenosine A_3_ receptors do not play a role in the regulation of renal fluid and transport[56], in distal nephron A6 cells, activation of adenosine A_3_ receptors promotes Cl^-^ secretion trough an increase in the influx of Ca^2+^[57]. Also, adenosine A_3_ receptors have been associated with mesangial cell apoptosis[24] and with direct[58] or transforming growth factor beta (TGB-β)-induced[59] expression of fibrosis markers in proximal tubule cells.

### STZ-induced diabetes altered the renal expression of adenosine receptors of the SHR

In our study, SHR with STZ-induced diabetes had a markedly higher expression of the adenosine A_1_ receptor in the DG when compared to the control SHR group. Although this effect (50% increase) did not reach statistical significance, it is in the opposite direction as that reported in normotensive Sprague-Dawley rats, where STZ-induced diabetes decreased adenosine A_1_ receptor immunostaining[21]. Although only speculative for now, since the adenosine A_1_ receptor is associated with glomerular constriction[60], this tendency to increase could represent an attempt to restraint intraglomerular pressure (and GFR). We have previously reported that these SHR-STZ rats show decreased SBP when compared with their SHR controls[61] but, even though this was associated with a decrease in renal cortical oxidative dysfunction, early diabetic renal damage was still evident, as indicated by increased GFR and proteinuria[61]. Overall, this suggests that, at least regarding adenosine regulation in the context of diabetes and hypertension, alterations in glomerular adenosine A_1_ receptors are triggered but are not enough to normalize renal hemodynamics. Interestingly, the increase in glomerular adenosine A_1_ receptor immunoreactivity would also decrease the blood output through the postglomerular afferent arterioles, compromising the already low blood supply to the renal medulla, thus aggravating the renal damage, which was confirmed by the clear presence of medullary oxidative stress[61].

Moreover, STZ-induced diabetes in SHR animals was associated with downregulation of the adenosine A_2A_ receptors in DG and PCT when compared to the SHR control group. Given the known vasodilator effects of adenosine A_2A_ receptor in the kidney[13, 61], the decreased glomerular expression in the DG of SHR-STZ animals (comparing to that of SHR controls) might favor a rise in intraglomerular pressure, thus contributing to the increased GFR that is observed in early SHR-STZ diabetic rats[61]. Adenosine can stimulate the Na^+^/K^+^-ATPase in PCT[60] through activation of adenosine A_2A_ receptors[62]. So, the downregulation of adenosine A_2A_ receptors observed in the PCT of SHR-STZ animals when compared with the SHR group might decrease adenosine A_2A_ receptor-mediated sodium reabsorption, promoting diuresis and natriuresis, which we have previously reported in these SHR-STZ animals[61].

The SHR-STZ group also showed lower expression of adenosine A_2A_ receptors in DCT when compared with the SHR control group. In the distal nephron, adenosine regulates Mg^2+^ homeostasis through decreased reabsorption via an adenosine A_1_-receptor mediated mechanism and increased reabsorption through activation of adenosine A_2A_ receptors[54]. We did not measure Mg^2+^ levels but published data and the results of this study are consistent with a role of adenosine as an important regulator of magnesium homeostasis, which is relevant from a translational perspective. Indeed, hypomagnesemia has been implicated in the progression of diabetic and hypertensive chronic kidney disease[63].

Most studies on the impact of diabetes on the expression and function of renal adenosine receptors have focused on the adenosine A_2B_ receptor and showed overexpression[21, 26]. Unexpectedly, our study showed similar expression of adenosine A_2B_ receptors between SHR-STZ rats and control SHR rats suggesting that in hypertensive animals this adenosine receptor subtype is not implicated in early STZ-diabetic nephropathy. To our knowledge, the immunolocalization of the adenosine A_2B_ receptor in the PCT has never been previously reported and its effects on this renal structure have not been studied yet. However, based in our observations, adenosine A_2B_ receptors might influence renal function in diabetes-associated with hypertension since its expression was almost abolished in SHR-STZ rats, although the difference was not statistically different from control SHR rats. The adenosine adenosine A_2B_ receptor has been reported to be relevant in the early stages of diabetic nephropathy for restraining mesangial cell growth[64]. However, it has also been implicated in later detrimental effects, namely renal fibrosis and glomerulosclerosis, through IL-6 formation[65] and the release of VEGF[21, 22] and TGF-β1[26].

In our experimental conditions, there was no difference in the expression of adenosine A_3_ receptors between SHR-STZ rats and control SHR rats, which suggests that adenosine A_3_ receptor-mediated mechanisms are non-operating in the early hyperfiltration conditions of diabetes associated with hypertension.

In conclusion, the four adenosine receptor subtypes (A_1_, A_2A_, A_2B_ and A_3_) are expressed in the kidney of SHR rats, although the distribution pattern is specific for each receptor subtype. Furthermore, STZ-induced diabetes in SHR rats affects their distribution mostly by downregulating the expression of A_2A_ receptors, which might be relevant for the development of early diabetes-associated hyperfiltration. Future studies will address whether endogenous or exogenous adenosine levels are relevant for the expression of renal adenosine receptors in the context of diabetes associated with hypertension.
